# Assessing Patient Perceptions of Virtual Reality Use for Pelvic Exams

**DOI:** 10.1089/jmedxr.2024.0045

**Published:** 2025-06-16

**Authors:** Katherine Moran Sweterlitsch, Eynav E. Accortt, Kenneth H. Kim, Melissa Wong, B.J. Rimel

**Affiliations:** ^1^ Department of Obstetrics and Gynecology, Cedars-Sinai Medical Center, Los Angeles, California, USA.; ^2^ Department of Informatics, Cedars-Sinai Medical Center, Los Angeles, California, USA.

**Keywords:** virtual reality, pelvic exam, anxiety, depression, trauma, pain

## Abstract

The objectives of this study were to evaluate patient willingness to use virtual reality (VR) for future pelvic exams and whether this proclivity is associated with a history of trauma and/or mental health concerns. This was an Institutional Review Board-approved acceptability pilot study performed at a single site with human participants. Trauma/toxic stress (Adverse Childhood Experiences ≥4), post-traumatic stress disorder (Impact of Events Scale ≥26), and anxiety (Overall Anxiety Severity and Impairment Scale ≥7) screenings were performed. Patients then attended a proctored VR experience and completed a modified Unified Theory of Acceptance and Use of Technology (mUTAUT) survey. Analysis was performed using descriptive statistics and logistic regression. Fifty percent (6/12) of patients indicated they would utilize VR during a future pelvic exam. Trauma/toxic stress, post-traumatic stress, and anxiety rates were 25%, 25%, and 58.3% respectively, but were not associated with device use willingness. The mUTAUT scores showed VR proclivity based on *facilitating conditions*, *social influence*, and *behavioral intentions*. Half of the patients would opt for VR during future pelvic exams, but no correlation was seen with anxiety, post-traumatic stress, or trauma/toxic stress.

## Introduction

Pain and anxiety are commonly experienced by patients undergoing medical interventions regardless of the clinical setting. Clinicians are working toward finding adjuvant or complementary therapies to reduce these negative experiences, such as music or animal therapy.^1–3^ Improvement in the patient experience is associated with not only increased adherence to therapy but also increased likelihood of participating in future interventions.^4,5^ Virtual reality (VR) is an emerging technology that has been assessed for its efficacy in decreasing symptoms of anxiety, distress, and pain among both adults and children in medical settings.^6–12^ This technology utilizes the power of distraction by shifting focus away from unpleasant stimuli in order to relieve both physical and psychological symptoms.^6^ The more directly engaged patients are with a VR interface at the time of the perceived negative intervention, such as through combined audio, visual, and interactive stimuli, the greater the benefit of the device due to the ability to activate multiple senses at once.^7,8^ This technology has been explored in various settings ranging from dressing changes to chemotherapy, all with positive results.^6,9,13^ However, research surrounding VR use within Obstetrics and Gynecology (OBGYN) is limited. When it has been studied in the field of OBGYN, VR has shown promising results including pain reduction during hysteroscopy, labor induction, intrauterine device (IUD) placement, and dilation and curettage.^8,13–18^ Additionally, VR has been shown to reduce preoperative anxiety in patients undergoing minor gynecological surgeries.^19,20^

Previous studies have focused mostly on pain and anxiety reduction, but have not explored patient perceptions regarding the use of VR during pelvic exams. There is even less understanding of the variation in preferences and the need for these services in patients who have had a traumatic experience in their past. Symptoms of anxiety, pain, embarrassment, and even fear are common surrounding pelvic exams so much so that patients may delay or even avoid examination altogether.^21^ Those who have experienced domestic or sexual violence are more likely to report adverse experiences with pelvic exams, such as discomfort, anxiety, stress, and increased pain.^22^ These physical exams place patients in not only vulnerable positions but also occur when they are undressed from the waist down. VR headsets often completely cover a person’s eyes with no way to see the outside world, essentially blindfolding the individual wearing the device. Trauma-informed care, which involves close provider communication, is important during vulnerable examinations in order to maintain a sense of safety for patients in addition to control.^23^ VR research often highlights positive results and does not adequately endorse some of the negative side effects of device use, such as detracting from the physician–patient interaction.^24,25^ A recently published study examined the use of VR during pelvic exams but only focused on pain and anxiety without further psychological assessment of the device’s impact in this setting.^26^ Therefore, patient perceptions of VR use during pelvic exams needs to be addressed first before wide implementation in this setting given the fundamental nature of these exams and how VR functions. We hypothesize that individuals with a history of trauma and/or mental health conditions will be more likely to have a perceived benefit from future VR use during pelvic exams. This study is one of the first to assess patient willingness to deploy VR as a future intervention during pelvic examination or procedures.

## Materials and Methods

This was an acceptability pilot study involving human subjects conducted at a single-site academic Gynecologic Oncology clinic at Cedars-Sinai Medical Center. This study was approved by the Cedars-Sinai Medical Center Institutional Review Board and was conducted in accordance with both ethical standards and institutional guidelines. Enrollment began in March 2023 and was completed in April 2023. All established clinic patients over the age of 18 who were English-speaking and able to give informed consent were eligible for the study. Patients who were new consults with a first-time visit during the study time frame were not included. Prior to their scheduled follow-up visits in the clinic, patients were screened via chart review for eligibility and called if they met the study criteria. Patients were excluded if use of VR would have been inappropriate, such as those individuals at risk for motion sickness, disorientation or seizures (history of epilepsy, dementia, sensitivity to flashing lights/motion, a medical condition predisposing to nausea/dizziness, etc.), or any injury to the eyes/face/neck that would make the use of the hardware either challenging or uncomfortable.^27,28^ Specifically in this study, if upon chart review, patients had a documented history of vertigo, seizures, epilepsy, Alzheimer’s disease, dementia, or face, eye, or neck surgery, they were excluded from the study. A total of 88 existing patients were scheduled for in-person clinic appointments during the study time frame, and 32 were determined to be eligible to participate based on chart review of their medical conditions. All of the eligible patients were then phone-screened for interest in the study and consented if willing to join. The most common reason for patients declining to participate after answering the screening call was that they did not want to stay longer than their scheduled visit. A total of 14 patients consented to participate and were then sent preliminary VR experience surveys using the Research Electronic Data Capture tool for data collection.^29^

### Pre-VR experience surveys

The pre-VR experience surveys included a basic demographic and intake form as well as screenings for anxiety, post-traumatic stress disorder (PTSD), and trauma history. Basic demographic information included the following: age, sexual orientation, gender identity, education level, race, ethnicity, and marital status. The intake form also inquired about baseline opinions regarding pelvic exams. Patients completed the Adverse Childhood Experiences (ACES), a 10-item survey used to measure childhood trauma and toxic stress.^30^ Total scores range from 0 to 10, with higher scores indicating more exposure to adverse childhood experiences. A score of 4 or greater on the ACES survey is an indicator of childhood trauma and associated toxic stress. The questionnaire assesses 10 types of childhood trauma, including physical abuse/neglect, verbal abuse, sexual abuse, emotional abuse/neglect, mental illness, incarcerated relative, domestic violence, substance use, and divorce.^30^ To assess current symptoms of PTSD, the Impact of Events Scale (IES), a 15-item Likert-type scale of 0–5, was used to measure event-specific distress.^31^ The IES has a score range from 0 to 75, and a score of 26 or greater on the scale identifies clinically elevated symptoms concerning for PTSD.^32^ Finally, to assess current symptoms of anxiety, the Overall Anxiety Severity and Impairment Scale (OASIS), a 5-item Likert-type scale of 0–4 self-reporting measure was employed.^33^ The OASIS has a score range of 0–20, and a score of 7 or greater indicates clinically elevated symptoms concerning for anxiety.^32^

### VR experience

Patients then individually presented to the clinic for an in-person VR experience with the Pico Google G2 4K headset. A single Pico Google G2 4K headset was donated by AppliedVR for the purpose of this study. During this time, patients wore the headset for a virtual experience but did not undergo a simultaneous pelvic exam for this study. Of the 32 patients who met the inclusion criteria, 14 gave consent for the study, but only 12 presented for the in-person session and completed all portions of data collection. At the beginning of the visit, the patients received an instructional orientation to the device from a study staff member who assisted with device placement, sound controls, and system navigation. Although the device itself has multiple experiences, all patients trialed the same experience for standardization. The patients were then directed to a 10-min guided meditation involving a visual and auditory experience with an interactive component of timed breathing. Specifically, patients were given commands by the meditation guide to direct the output force of their timed breathing at various structures to rebuild a dismantled shape in a peaceful natural setting. The study staff member remained silently with the patient throughout the entire VR experience for any troubleshooting. Participants were instructed that they were allowed to abort the VR experience for any reason at any time.

### Post-VR experience surveys

Once the in-person VR session ended, patients were directed to complete one final post-experience survey. The final survey consisted of a modified version of the Unified Theory of Acceptance and Use of Technology (UTAUT).^34–36^ The UTAUT model was originally designed to reveal acceptance, intentions, and usage behavior for information technology but has been adapted to assess additional technologies.^34–40^ This model examines the acceptance of the technology of interest based on the following dimensions: *performance expectancy*, *effort expectancy*, *perceived enjoyment*, *user attitudes*, *behavioral intentions*, *social influence*, and *facilitating conditions*. For this study, the model was modified by creating pertinent questions to capture the essence of patient perceptions surrounding the use of VR for future pelvic examination, similarly to a study conducted by Shao et al.^36^ The focus of this previous study was to assess VR use for socialization of elderly patients, thus the UTAUT survey included questions surrounding entertainment and communication with others.^36^ As detailed in Figure 1, the questions for the purpose of this study were redesigned for study relevance in a similar fashion. The modified UTAUT (mUTAUT) was then administered in survey form for patients to complete. On this final survey, patients were then asked to answer “yes” or “no” as to whether they would select to use VR during a future pelvic exam, in addition to being given a free-form space to provide any comments.

**FIG. 1.**
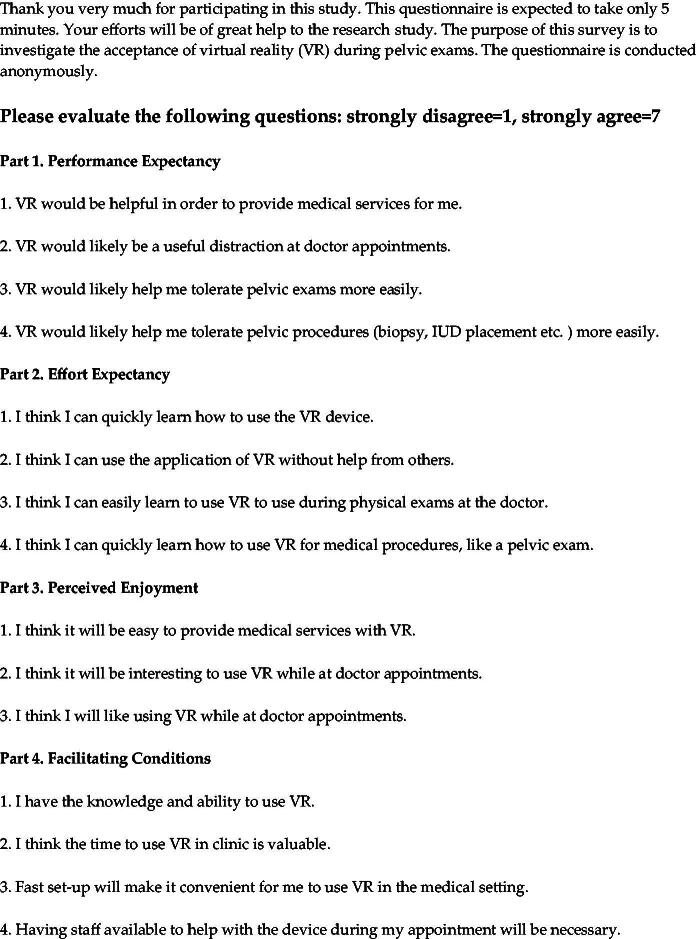
Modified Unified Theory of Acceptance and Use of Technology.

**FIG. 1.**
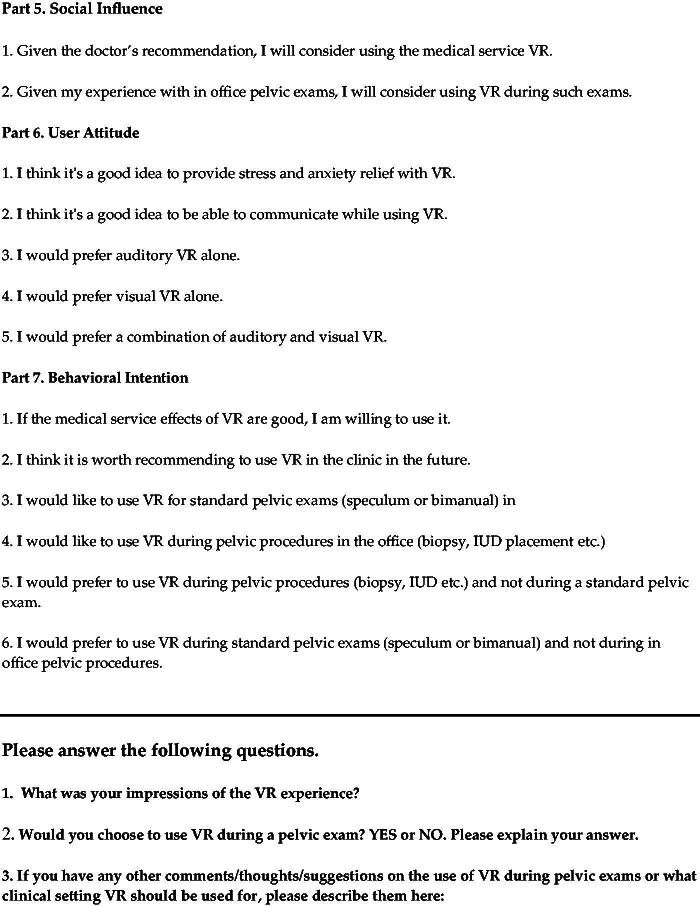
(Continued)

### Statistical analysis

For the results of the survey data, continuous variables were reported as median and interquartile range. Scores above the standardized cutoff score criteria for OASIS, IES, and ACES were calculated and reported as a proportion. Responses to the post-VR experience mUTAUT survey were reported as mean and standard deviation. Internal consistency of these scores was assessed using Cronbach’s α for reliability. Univariate analysis was performed to assess whether the mean mUTAUT scores were associated with patients’ desired use of VR during a pelvic exam.^41^ Multivariate analysis was performed to assess whether the cutoff scores for the OASIS, IES, and ACES were associated with patients’ desired use of VR during a pelvic exam. Statistical analysis was performed using the Software Package for Statistics and Simulation (IBM SPSS version 27; IBM Corp., Armonk, NY).

## Results

A total of 12 patients met the inclusion criteria and completed all portions of the study (Table 1). The average age was 52.8 (±12.8), and all patients self-reported as White/Caucasian, with the majority self-reporting as non-Hispanic/non-Latinx (9, 75%). A total of 10 (83.3%) patients were heterosexual/straight, and 2 (16.7%) were homosexual/gay/lesbian. A total of 11 patients were cisgender women (91.7%), and 1 was a transgender man (8.3%). The majority of patients were married (7, 58.3%) and had a bachelor’s degree (6, 50%).

**Table 1.** Demographics of Patients

Characteristic	*n* = 12
Age (mean years ± SD)	52.8 ± 12.8
Race	
White/Caucasian	12 (100%)
Ethnicity	
Hispanic/Latinx	3 (25.0%)
Non-Hispanic/non-Latinx	9 (75.0%)
Sexual orientation	
Heterosexual/straight	10 (83.3%)
Homosexual/gay/lesbian	2 (16.7%)
Bisexual	0
Pansexual	0
Queer	0
Other	0
Gender identity	
Cis gender woman	11 (91.7%)
Transgender man	1 (8.3%)
Gender queer	0
Two spirit	0
Gender diverse	0
Intersex	0
Other	0
Marital status	
Single/never married	3 (25.0%)
Married	7 (58.3%)
Divorced/separated	2 (16.7%)
Highest education level	
High school or GED	3 (25.0%)
Bachelor’s degree	6 (75.0%)
Master’s degree	3 (25.0%)

GED, General Educational Development; SD, standard deviation.

The median ACES score was 1 (interquartile range [IQR] = 0–5.0), with 25% of patients with scores over 4 indicating a history of trauma (Table 2). The median IES score was 18.5 (IQR = 6.5–33.3), with 25% of patients with scores over 26 indicating symptoms concerning for PTSD. The median OASIS score was 7.5 (IQR = 5.0–10.3), with 58.3% of patients with scores over 7 indicating symptoms concerning for anxiety. Baseline perceptions of pelvic exams were as follows: necessary (9, 75%), uncomfortable (7, 58.3%), embarrassing (2, 16.7%), painful (3, 25%), tolerable or not bothersome (3, 25%), or anxiety inducing (6, 50%) (Table 2).

**Table 2.** Pre-Virtual Reality Experience Survey Results

Characteristic	*n* = 12
Mental health screening	
ACES (median, IQR)	1 (0–5.0)
ACES ≥4	25.0%
IES (median, IQR)	18.5 (6.5–33.3)
IES ≥26	25.0%
OASIS (median, IQR)	7.5 (5.0–10.3)
OASIS ≥7	58.3%
Baseline thoughts regarding pelvic exams (*n*, %)	
Necessary	9 (75.0%)
Uncomfortable	7 (58.3%)
Embarrassing	2 (16.7%)
Painful	3 (25.0%)
Tolerable/not bothersome	3 (25.0%)
Anxiety inducing	6 (50.0%)

The ACES questionnaire is an assessment evaluating childhood trauma/toxic stress with scores ≥4 determined to be associated with adult mental and physical health problems. IES scores of ≥26 are associated with risk for current post-traumatic stress disorder. OASIS scores ≥7 indicate risk for current anxiety.

ACES, Adverse Childhood Experiences; IES, Impact of Events Scale; IQR, interquartile range; OASIS, Overall Anxiety Severity and Impairment Scale.

**FIG. 2.**
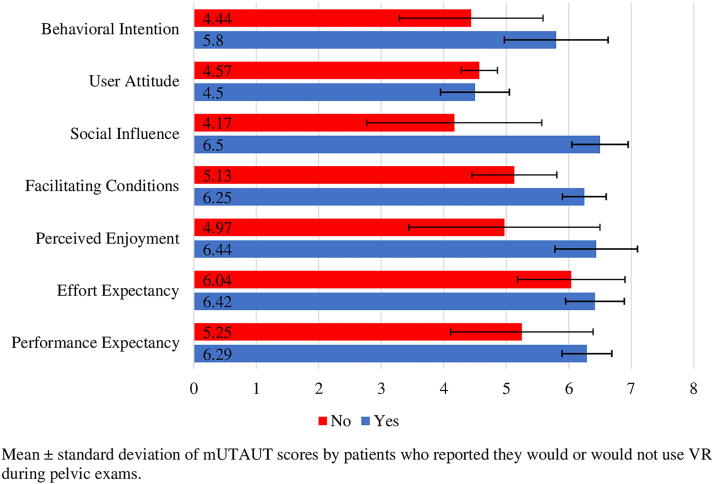
Differences in willingness to use VR based on mUTAUT average scores. mUTAUT, modified Unified Theory of Acceptance and Use of Technology; VR, virtual reality.

For the primary outcome of the study—patient willingness to use VR during a pelvic examination—the responses were mixed: six (50%) were in favor of device use and six (50%) were against it. For three components of the mUTAUT survey, Cronbach’s α was greater than 0.7, indicating high reliability (Table 3). A statistically significant observation for VR preference was noted for mean mUTAUT scores for the following categories: *facilitating conditions*, *social influence*, and *behavioral intention* (Fig. 2). Patients who indicated “yes” to future VR use during a pelvic exam on the final questionnaire had statistically higher scores for *facilitating conditions* (6.25 ± 0.35 “yes” vs. 5.13 ± 0.68 “no”, *p* < 0.01), *social influence (*6.50 ± 0.45 “yes” vs. 4.17 ± 1.54 “no”, *p* < 0.01), and *behavioral intentions* (5.80 ± 0.83 “yes” vs. 4.44 ± 1.15 “no”, *p* < 0.04).

**Table 3.** Descriptive Analysis of the Modified Unified Theory of Acceptance and Use of Technology

Characteristic	No. of questions	Cronbach’s α	Mean	SD
Performance expectancy	4	0.680	5.77	0.98
Effort expectancy	4	0.311	6.23	0.69
Perceived enjoyment	3	0.871	5.71	1.36
Facilitating conditions	4	0.205	5.69	0.78
Social influence	2	0.857	5.33	1.63
User attitude	5	0.170	4.53	0.42
Behavioral intention	6	0.796	5.13	1.19

To assess for a relationship between VR use acceptability for pelvic exams and history of trauma and/or risk of mental health conditions, cutoff scores for ACES, IES, and OASIS were compared with willingness to use the device (Table 4). We found no statistically significant difference between the cutoff score for ACES, IES, or OASIS and willingness to use VR. Interestingly, all patients with an ACES score greater than 4, and two-thirds of patients with an IES score greater than 26, would choose to use VR. Of the patients that had an OASIS score greater than 7, more than half would choose not to use VR during a pelvic exam.

**Table 4.** Differences in Willingness to Use Virtual Reality Based on Adverse Childhood Experiences, Impact of Events Scale, and Overall Anxiety Severity and Impairment Scale Scores

Characteristic	Yes	No	*p*
ACES total ≥4 (childhood trauma/toxic stress risk)	100%	0	0.09
IES ≥26 (PTSD risk)	66.7%	33.3%	0.50
OASIS ≥7 (anxiety risk)	42.9%	57.1%	1.00

PTSD, post-traumatic stress disorder.

Patient summative comments regarding the device are shown in Table 5 and consist of both positive and negative views. A summary of positive comments includes the following endorsements for VR: relaxing, beautiful, easy, calming, enjoyable, and a pleasant distraction. A summary of the negative comments includes the following: exams are too short to use the device, and it would take away from the provider interaction/engagement causing more stress.

**Table 5.** Patient Summative Comments

Positive comments
“It was very relaxing and beautiful. I was focusing more on how beautiful it was than my breathing, but definitely can see where the experience would help people with anxiety during pelvic exams.”
“Really enjoyed how easy it was to get started—totally forgot where I was by the time the session ended! I think this is how I could get through a pelvic exam.”
“I personally can tolerate the discomfort of a quick pelvic exam and like to use the extra few minutes to talk to my doctor. For others who might find it very uncomfortable I think VR is a great option.”
“This is a great tool for calming and distraction.”
“I would definitely use VR especially during a biopsy.”
“Being relaxed during a pelvic exam is important, so I feel VR can help with achieving that.”
“Enjoyable; it took me out of my head. One with tasks would be most effective.”
“Even though I don’t find these exams uncomfortable it would be nice to have such a pleasant distraction.”
Negative comments
“I would like to interact with my doctor. I like being told exactly what is happening.”
“I think depends on where you are at in your experience. I had never seen an OBGYN until I had cancer and I was nervous at first but now I am 15 months in remission so exams are not a problem anymore. I think wearing the device would take away from hearing the provider communicate and tell you when to push or when you would feel pain.”
“I think the procedure itself [pelvic examination] is a short time procedure. Perhaps no time to set up and use?”
“I typically do not experience anxiety during exams and I want to remain engaged with the doctor, however, I would consider if I was feeling anxious about a particular procedure.”
“Pelvic exams stress me out, and in order to cope with them, I’m intensely focused on the doctor and what she’s saying or doing. I’m concerned that having the VR would potentially cause me more stress because it would inhibit my ability to listen to the doctor.”

OBGYN: Obstetrics and Gynecology.

## Discussion

We found that 50% of patients stated they would choose to use VR during a future pelvic exam, but there was no difference seen for individuals with a trauma history or mental health conditions. Although patients scored similarly regarding *perceived enjoyment*, *social influence*, and *behavioral intention* for the device, the factors most associated with willingness to use VR were *facilitating conditions*, *social influence*, and *behavioral intentions*. Given the small number of participants, the results of this study are not meant to guide decision making surrounding which patients are candidates for VR, but are to highlight that VR is not for all patients. Further studies are needed to uncover those who would benefit the most from this technology.

Traditionally, pelvic exams are employed for both routine screening and to investigate new symptoms. Exams often include both a visual inspection with a speculum in addition to a bimanual exam, sometimes with a rectovaginal assessment, in order to evaluate internal organs. These exams, however, have been associated with high rates of embarrassment, anxiety, fear, and pain for patients.^21^ Factors associated with an increased risk of these concerns include patient age, provider gender, history of sexual trauma, and previous exam with a negative experience.^21^ Although no statistically significant difference was revealed for anxiety, PTSD, and trauma/toxic stress with VR propensity in this study, many survey respondents gave positive feedback in regard to VR use for pelvic exams in the future. Most of the patients reported they believe the device offered a calming, relaxing, and distracting experience. The most commonly endorsed concern, though, was that the device would limit the interaction with their provider during the exam. There are some newer headsets that use “passthrough” technology to allow a user to be aware of their actual surroundings while still seeing virtual images, which could combat this concern.^42,43^ Of note, this study did find that all patients with a history concerning for trauma (ACES score ≥4) and two-thirds of those who screened positive for PTSD (IES scores ≥26) indicated they would choose to use the device for pelvic exams, while patients with symptoms of anxiety (OASIS score ≥7) would not. This suggests that VR may be appropriate for some but not all patient populations, specifically regarding a psychological or trauma history.

### Findings in the context of previous literature

Prior VR randomized controlled trials in Gynecology have compared pre- and post-procedure anxiety and/or pain during outpatient procedures and have yielded inconsistent findings.^14–18^ For dilation and curettage or hysteroscopy, VR was shown to be an effective tool for reducing both anxiety and/or pain.^14,18^ For IUD placement on the contrary, studies have shown mixed results for the ability of VR to help with the reduction of pain and anxiety.^15–17^ This highlights that the use of VR is not a panacea for all exam discomfort nor does every patient benefit equally.

The mUTAUT revealed VR proclivity was associated with three arms of the model: *facilitating conditions*, *social influence of others*, and *future behavioral intentions*. *Facilitating conditions* reveal the overall perception an individual has of how conveniently a technology can be adapted for its intended purpose.^35–37^ Factors that influence *facilitating conditions* include the reliability of resources, as well as support and infrastructure for the technology to exist. As previously mentioned, this study showed a statistically significant difference between *facilitating conditions* and device proclivity, suggesting that if VR was to be used for pelvic exams, patients believe it would be easy to implement for this purpose. The *social influence* arm of the model aims to uncover if a person’s desire to use a technology can be persuaded by others.^35,36^ A statistically significant difference between those who would choose to use the device during a pelvic exam and *social influence* was noted. Willingness to use a new device in a vulnerable setting may be attributed to the degree of importance an individual places on the perception of others, such as a trusted physician or peers, who are making the recommendation.^44^ Finally, *behavioral intention* reflects the probability that an individual will engage with a technology for its intended use.^36,37^ The mUTAUT model showed a statistically significant difference between *behavioral intention* and willingness to use the VR device for future pelvic exams. Although there was not 100% agreement among study patients’ proclivity for VR use for future pelvic exams, this element of behavioral intention shows that people think it may be beneficial for this purpose, if not for themselves at this time.

### Strengths and limitations

One of the major strengths of this pilot study was the analysis of patient perceptions regarding VR use in a medical setting rather than a direct measure of pain or anxiety reduction. It is also the first, to our knowledge, to assess the acceptability and psychological impact of this technology during pelvic exams. Several limitations should be acknowledged. First, although 32 patients met the inclusion criteria, only 12 completed all aspects of the study. This small sample size is not representative of the general population. However, even with the small numbers, we did see a variety of questionnaire responses highlighting the different opinions surrounding VR, indicating that it may not be appropriate for everyone. Second, patients were from a single clinic, and further studies should incorporate patients from additional subsets of OBGYN that use pelvic exams. Last, this study focused on select mental health conditions and does not intend to be representative of all diagnoses that may make a patient more likely to have negative experiences with pelvic exams.^42–44^

### Future directions

Although not all patients wholeheartedly endorsed they would opt to use this technology during a future pelvic exam, this study highlights that certain subsets of patients may indeed benefit from this technology, but further work is needed to identify such individuals. Future work may also consider differential effects within transgender populations. Transgender males or nonbinary patients may experience heightened dysphoria with genital examination, which unfortunately can lead to avoidance of routine care.^45,46^ By contrast, pelvic exams may in fact be gender affirming for transfeminine patients.^46^ In the present study, one patient identified as a transgender male and reported that, “I think this [VR] is how I could get through a pelvic exam” (Table 5).

In conclusion, half of the patients would opt for VR during a future pelvic exam, but no difference was seen for individuals with current symptoms of anxiety, PTSD, or a history of trauma. Although no difference was seen with the studied conditions, evidence suggests there may be an ideal patient population that would desire VR to help improve routine exams. Future studies are needed to explore VR use during pelvic exams in order to find the appropriate population and setting for use before wide implementation.

## Abbreviations Used

ACES: Adverse Childhood Experiences
IES: Impact of Events Scale
mUTAUT: modified Unified Theory of Acceptance and Use of Technology
OASIS: Overall Anxiety Severity and Impairment Scale
PTSD: post-traumatic stress disorder
UTAUT: Unified Theory of Acceptance and Use of Technology
VR: virtual reality
